# The Recombinant Sea Urchin Immune Effector Protein, rSpTransformer-E1, Binds to Phosphatidic Acid and Deforms Membranes

**DOI:** 10.3389/fimmu.2017.00481

**Published:** 2017-05-12

**Authors:** Cheng Man Lun, Robin L. Samuel, Susan D. Gillmor, Anthony Boyd, L. Courtney Smith

**Affiliations:** ^1^Department of Biological Sciences, George Washington University, Science and Engineering Hall, Washington, DC, USA; ^2^Department of Chemistry, George Washington University, Science and Engineering Hall, Washington, DC, USA

**Keywords:** Sp185/333, echinoderm, innate immunity, conformational plasticity, liposomes, lipid clusters

## Abstract

The purple sea urchin, *Strongylocentrotus purpuratus*, possesses a sophisticated innate immune system that functions without adaptive capabilities and responds to pathogens effectively by expressing the highly diverse *SpTransformer* gene family (formerly the *Sp185/333* gene family). The swift gene expression response and the sequence diversity of *SpTransformer* cDNAs suggest that the encoded proteins have immune functions. Individual sea urchins can express up to 260 distinct SpTransformer proteins, and their diversity suggests that different versions may have different functions. Although the deduced proteins are diverse, they share an overall structure of a hydrophobic leader, a glycine-rich N-terminal region, a histidine-rich region, and a C-terminal region. Circular dichroism analysis of a recombinant SpTransformer protein, rSpTransformer-E1 (rSpTrf-E1) demonstrates that it is intrinsically disordered and transforms to α helical in the presence of buffer additives and binding targets. Although native SpTrf proteins are associated with the membranes of perinuclear vesicles in the phagocyte class of coelomocytes and are present on the surface of small phagocytes, they have no predicted transmembrane region or conserved site for glycophosphatidylinositol linkage. To determine whether native SpTrf proteins associate with phagocyte membranes through interactions with lipids, when rSpTrf-E1 is incubated with lipid-embedded nylon strips, it binds to phosphatidic acid (PA) through both the glycine-rich region and the histidine-rich region. Synthetic liposomes composed of PA and phosphatidylcholine show binding between rSpTrf-E1 and PA by fluorescence resonance energy transfer, which is associated with leakage of luminal contents suggesting changes in lipid organization and perhaps liposome lysis. Interactions with liposomes also change membrane curvature leading to liposome budding, fusion, and invagination, which is associated with PA clustering induced by rSpTrf-E1 binding. Longer incubations result in the extraction of PA from the liposomes, which form disorganized clusters. CD shows that when rSpTrf-E1 binds to PA, it changes its secondary structure from disordered to α helical. These results provide evidence for how SpTransformer proteins may associate with molecules that have exposed phosphates including PA on cell membranes and how the characteristic of protein multimerization may drive changes in the organization of membrane lipids.

## Introduction

**Figure 1:**
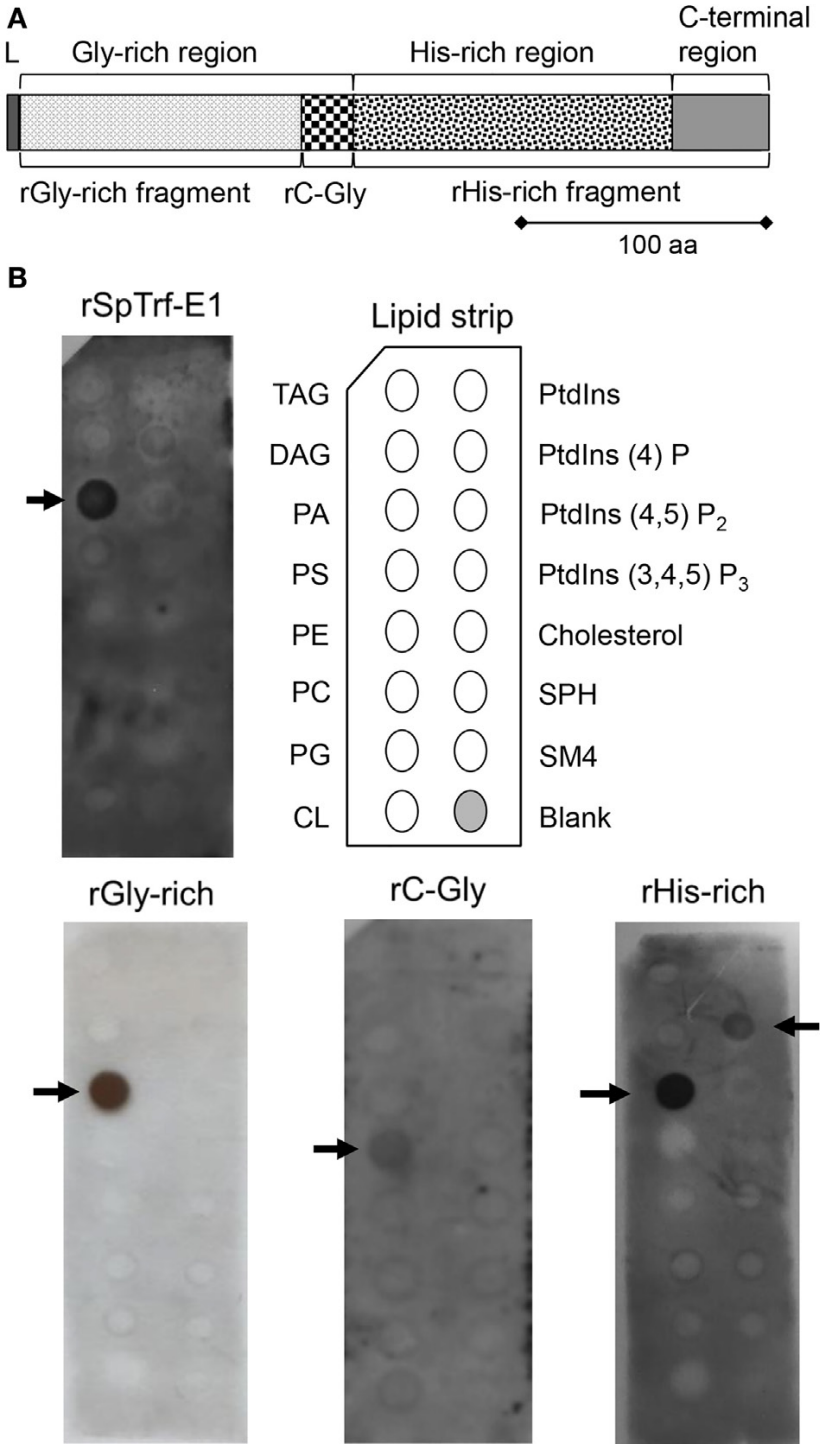
**Recombinant SpTransformer protein, rSpTransformer-E1 (rSpTrf-E1) and the recombinant fragments bind to lipids**. **(A)** The protein structure of rSpTrf-E1 shows four regions; the N-terminal leader (L), the Gly-rich region, the His-rich region, and the C-terminal region. Four recombinant proteins are evaluated for their lipid binding characteristics using a lipid-embedded nylon strip **(B)**; the full-length rSpTrf-E1 protein, the rGly-rich fragment, the C-terminal end of the Gly-rich region called rC-Gly, and the rHis-rich fragment. **(B)** rSpTrf-E1, the rGly-rich and the rHis-rich fragments bind to PA. The rHis-rich fragment also binds to PtdIns(4)P. The rC-Gly fragment binds only to PS. Arrows indicate the phospholipids to which the proteins bind. The nylon strip is embedded with spots of TAG, trisacylglyceride; DAG, diacylglycerol; PA, phosphatidic acid; PS, phosphatidylserine; PE, phosphatidylethanolamine; PC, phosphatidylcholine; PG, phosphatidylglycerol; CL, cardiolipin; PtdIns, phosphatidylinositol; PtdIns(4)P, phosphatidylinositol-4-phosphate; PtdIns(4,5)P_2_, phosphatidylinositol 4,5 bisphosphate; PtdIns(3,4,5)P_3_, phosphatidylinositol 3,4,5 triphosphate; SPH, sphingomyelin; SM4, 3-sulfogalactosylceramide; cholesterol.

The genome of the California purple sea urchin (*Strongylocentrotus purpuratus)* has a number of large immune gene families that are quite complex (1–3). One of the families, *SpTransformer* (*SpTrf*, formerly *Sp185/333*), is unique to sea urchin species that are members of the euechinoidea subclass and show no homology to gene families in other organisms including the cidaroidea subclass of echinoids. The *SpTrf* gene family has been estimated to have ~50 members, and the genes have two exons that encode the leader and the mature protein (4–9) that respond with swift increases in expression upon challenges from microbes and pathogen-associated molecular patterns (PAMPs) (4, 10, 11). Alignments of genes and transcripts require the insertion of artificial gaps in the second exon that defines 25–27 blocks of sequences known as *elements* (5). The presence and absence of elements create different mosaics of elements that are repeatedly identified and called *element patterns* [(4, 5, 11), reviewed in Ref. (8)]. Despite the sequence diversity of the genes and transcripts, the encoded proteins have a generic structure that is composed of an N-terminal leader, a glycine-rich (Gly-rich) region with an arginine–glycine–aspartic acid motif, a histidine-rich (His-rich) region, and a C-terminal region (Figure 1A) (4, 12). The diversity of element patterns, plus putative editing of the *SpTrf* mRNAs (13) that introduces missense sequence and early stop codons produces a wide range of deduced SpTrf proteins of 4–55 kDa (4, 11). An evaluation of the native (nat)SpTrf proteins in one sea urchin suggests that it can express up to 260 different variants and that the native proteins appear unexpectedly large on Western blots relative to the deduced protein size predictions (14–16). Increases in size are likely the result of multimerization of natSpTrf proteins that is induced by isolation and processing, which can also be induced for a recombinant (r)SpTrf-E1 protein (originally called rSp0032) after isolation from *E. coli* and in the absence of other sea urchin proteins (12, 14). Once multimerized, SpTrf proteins, whether native or recombinant, cannot be separated to monomers [see supplemental materials in Ref. (16)] and once bound cannot be dissociated from the marine bacteria, *Vibrio diazotrophicus*, or Baker’s yeast, *Saccharomyces cerevisiae* (12). Furthermore, recombinant SpTransformer protein, rSpTransformer-E1 (rSpTrf-E1), binds tightly to lipopolysaccharide (LPS), β-1,3-glucan, and flagellin and once bound cannot be dissociated from one PAMP for rebinding to another.

The deduced amino acid composition of the SpTrf proteins indicates two major regions within the proteins, the Gly-rich and His-rich regions, that have been predicted to be functionally different (4, 11). The first functional evaluation of rSpTrf-E1 plus three discrete recombinant fragments, the rGly-rich fragment, the C-terminal end of the Gly-rich region called the rC-Gly, and the rHis-rich fragment (Figure 1A) demonstrated that rSpTrf-E1 has restricted binding to a subset of bacterial species (12). However, the recombinant fragments all show expanded bacterial binding suggesting differences in the activities of the separated fragments. The rC-Gly fragment is consistently multimerized upon isolation and likely mediates multimerization of full-length natSpTrf proteins. In the absence of the rC-Gly fragment, neither the rGly-rich nor the rHis-rich fragments show multimerization either upon isolation, after storage, or upon binding targets. When using yeast as a binding target, rSpTrf-E1 partially competes with the rGly-rich fragment and fully competes with the rHis-rich fragment, indicating that both ends of the protein bind to yeast, but that the rGly-rich fragment has expanded binding activities. This result is noteworthy because mRNA editing that tends to occur prior to immune challenge (16) produces truncated proteins that consist only of the Gly-rich region in which the expanded binding activity may act in immune surveillance in the sea urchin (12).

Recombinant SpTransformer protein, rSpTransformer-E1 (rSpTrf-E1), is an intrinsically disordered protein (IDP) in which monomers undergo secondary structural transformation from disordered to 78–95% α helical in sodium dodecyl sulfate (SDS), trifluoroethanol (TFE), or LPS (17). This secondary structural transformation is the basis for the new name of SpTrf proteins and rSpTrf-E1 has an E1 element pattern, hence the name extension. For details on element patterns and naming convention, see Ref. (4, 5, 9, 11, 18). Based on the overall structural similarities among the SpTrf proteins and bioinformatic predictions that all may be IDPs as suggested by amino acid sequences, we have speculated that many may have similar transforming capabilities and show structural changes that are induced by binding targets. The rHis-rich and rGly-rich fragments also show changes in secondary structural conformation; however, the changes are unexpected relative to results for rSpTrf-E1. The rGly-rich and rHis-rich fragments are 15–30% α helical in phosphate buffer rather than disordered like rSpTrf-E1. In the presence of SDS, TFE, or LPS, the fragments either enhance their α helical structure or switch to β strand structure (17). These results suggest that the Gly-rich and His-rich regions within rSpTrf-E1 likely interact and influence both the specificity of the target to which they bind and the subsequent folding upon binding to a target.

Native SpTrf proteins are present within the perinuclear vesicles of all types of phagocytes and are present on the surface of small phagocytes, although the percentage of cells that express the proteins is variable among animals (14, 19). HeTransformer proteins (HeTrf, formerly He185/333) have also been noted in association with vesicle membranes and with plasma membranes of gut-associated amoebocytes (an alternative term for phagocytes) from another sea urchin species, *Heliocidaris erythrogramma* (20, 21). Yet, the association of Trf proteins from both sea urchin species with cell membranes is not predicted from their deduced amino acid sequences; there are no recognizable transmembrane regions and no predicted conserved motifs for glycophosphatidylinositol linkages (4, 14, 21). To understand this association, we investigated possible interactions of rSpTrf-E1 and the recombinant fragments with phospholipids and identified specific binding to phosphatidic acid (PA) by the full-length protein and the rGly-rich and rHis-rich fragments. In addition, the rHis-rich fragment also binds to phosphatidyl inositol 4 phosphate [PtnIns(4)P], although with lower affinity. rSpTrf-E1 binds to liposomes that are composed of 10% PA and 90% phosphatidylcholine (10% PA:PC) and transforms from disordered to ~70% α helical in the presence of PA. rSpTrf-E1 induces changes in membrane curvature of 10% PA:PC liposomes, which show budding, invagination, and fusion that is associated with PA clustering. rSpTrf-E1 induces leakage of materials captured within liposome lumens, and longer incubations with 10% PA:PC liposomes result in PA extraction from the membranes. We speculate that accessible phosphate groups may be a binding target and that this may be a mechanism by which a subset of natSpTrf proteins may interact with coelomocyte membranes in sea urchins and perhaps may aid in initiating membrane curvature through PA clustering leading to phagocytosis of bacteria.

## Materials and Methods

### Expression, Isolation, and Purification of natSpTrf proteins, rSpTrf-E1, and the Recombinant Fragments

The expression, isolation, and purification of rSpTrf-E1, and the three recombinant fragments from *E. coli* were performed as described (12). Nickel affinity was used to isolate natSpTrf proteins (Ni-natSpTrf) according to Sherman et al. (16) with an additional step using anti-SpTrf (formerly anti-Sp185/333) antibodies linked in an affinity column according to Lun et al. (12). Following isolation by Ni-affinity, Ni-natSpTrf, rSpTrf-E1, and the recombinant fragments were verified by analysis of flow-through and elution fractions on Any KD™ Mini-PROTEAN^®^ TGX precast gels (Bio-Rad Laboratories, Inc.) that were electrophoresed for 20 min at 300 V and constant voltage. Two precast gels were run simultaneously; one was processed for Western blot evaluation with anti-SpTrf antibodies, and the other was stained with Biosafe Coomassie stain (Bio-Rad Laboratories) as described (12).

### Phospholipid Nylon Strip Binding

Nylon strips with embedded phosphatidylinositol (PtdIns) lipids and other phospholipids (100 pmol per spot; Echelon Biosciences) were pre-incubated in blocking buffer [3% bovine serum albumin (BSA; w/v; fatty acid free) in standard phosphate-buffered saline (PBS) pH 7.4 with 0.1% Tween-20 (PBST)] for 2 h at room temperature (rt) on a rocking platform. rSpTrf-E1 or recombinant fragments (~20 nM) were incubated with a lipid-embedded strip in fresh blocking buffer for 2 h at rt with rocking. Unbound proteins were removed with three washes of PBST. Strips were incubated for 2 h at rt with primary antibodies composed of three polyclonal rabbit anti-SpTrf antibodies [anti-SpTrf-66, -68, and -71; 1:3,500 dilution (14, 15)] in blocking buffer, washed, and post-incubated with goat anti-rabbit IgG conjugated to horseradish peroxidase (GαRIg-HRP; 1:7,000 dilution in blocking buffer; Thermo Scientific Pierce) for 1 h at rt with rocking. Antibody–protein complexes on washed strips were visualized by incubation with enhanced chemiluminescence Western blotting substrate (Thermo Scientific Pierce) and exposed to autoradiography film (MidSci). Experiments were performed at least three times to confirm binding between rSpTrf-E1 or the recombinant fragments and the phospholipids. Negative controls omitted either rSpTrf-E1, the recombinant fragments, or the primary antibodies.

### Liposome Preparation

Small unilamellar vesicles (SUVs; <100 nm) were prepared according to Kessler et al. (22) with modifications using varying mixtures of lipid concentrations including 100, 95, 90, and 80% of 1,2-dioleoyl-*sn*-glycero-3-phosphocholine (dioleoyl PC) and a corresponding 0, 5, 10, and 20% of 1,2-dioleoyl-*sn*-glycero-3-phosphate (PA; Avanti Polar Lipids, Inc.). For fluorescence resonance energy transfer (FRET) assays (see below), 1,1′dioctadecyl-3,3,3′,3′-tetramethylindocarbocyanine perchlorate (DiI) was added to the lipid mixture at a mass ratio of 1:800 (DiI:PC). The lipid mixture was dried under nitrogen in test tubes that were cleaned with base bath (ethanol with potassium hydroxide solution) and rinsed several times with distilled water. Lipids were dissolved in chloroform and rotor-evaporated under nitrogen for 30 min followed by vacuum desiccation (Bel-Art Products) to remove all organic solvents. Sucrose (2%) in PBS at 80°C was added to the desiccated lipids and vortexed for 30 s at rt until the solution became opaque. The sucrose–lipid mixtures were incubated at 80°C in an Isotemp oven (Thermo Fisher Scientific) for 15 min followed by vortexing for 30 s, which was repeated twice. Lipids were resuspended in distilled water to multilamellar vesicles and converted to SUVs by bath sonication for 1–2 h with an UltraSonic Cleaner FS30H (Thermo Fisher Scientific). The SUV size range was determined using dynamic light scattering on a Beckman Coulter N5 submicron particle size analyzer with a 1-cm path length cuvette with a 30-min equilibrium time at rt with light scattering angle of 90°. The average size from three repetitions was evaluated and reported as the size of the SUVs.

Large unilamellar vesicles (LUVs; ~100–1,000 nm) were generated by mixing lipids in a ratio of 10% PA to 90% PC in chloroform followed by initial drying under nitrogen gas followed by a secondary drying step under vacuum for 2 h. Vesicles were rehydrated in standard PBS with 10 mM 8-aminonaphthalene-1,3,6-trisulfonic acid disodium salt (ANTS; dye) and 15 mM *p*-xylene-Bis-pyridinium bromide (DPX; quencher). Liposomes were allowed to swell for 5 min before vortexing for 30 s, followed by heating to 45°C and undergoing five cycles of freeze/thaw using a dry ice—ethanol bath for 3 min per cycle, and ending with an incubation at 45°C for 5 min. Excess dye and quencher surrounding the loaded liposomes were removed by gel filtration through a Sephadex^®^ G-25 Medium (Sigma-Aldrich) column. This procedure generated liposomes of 90% LUVs (50–1,000 nm) and 10% SUVs as measured by dynamic light scattering (Wyatt Technologies). The concentration of LUVs was determined according to Antimisiaris (23).

Giant unilamellar vesicles (GUVs, >1 μm) were synthesized using electroformation according to Angelova and Dimitrov (24) with modifications from Kessler et al. (22). The lipids were combined in 1:9 M ratio (v:v) of PA:PC in a chloroform and methanol solvent. The fluorescent dye 1,1′-dioctadecyl-3,3,3′,3′-tetramethylindodicarbocyanine perchlorate (DiD) was incorporated into the lipid bilayers for an overall 0.08 mol% of DiD to lipid. For PA clustering detection, 6% 1-oleoyl-2-(6-[(7-nitro-2-1,3-benzoxadiazol-4-yl)amino]hexanoyl)-*sn*-glycero-3-phosphate (NBD-PA) and 4% unlabeled PA were mixed with 90% PC. Lipids and dyes were mixed thoroughly to homogeneity and 10 µl of the mixed sample was coated onto two platinum wire electrodes (1.2 mm diameter). The electrodes with the lipid mixture were placed inside a vacuum desiccator to complete the solvent evaporation followed by emersion in a non-electrolyte buffer solution of 2% (w/v) sucrose. For microscopy imaging, 0.167 µM dextran labeled with Alexa Fluor^®^ 488 (dextran-488, 3,000 MW, Anionic; Thermo Scientific Invitrogen) was added to the sucrose solution. Electrodes were connected to a waveform generator (Hewlett Packard) and incubated at 80°C during the electroformation procedure that started at 0.7 V with a frequency of 10 Hz, followed by stepwise voltage increases of 0.05 V every 5 min to 1.4 V, which was maintained for 3 h. Vesicles were separated from the electrodes by a final step of 0.6 V and 4 Hz, and sample cells were allowed to cool slowly to rt overnight. To separate the vesicles from the dextran-488 in solution that was not incorporated into the vesicles lumens, 300 µl of vesicles in solution were mixed with 100 µl of sugar solution (1.8% sucrose, 0.2% glucose) and spun at 15.8 × 10^3^ × *g* for 10 min in a microfuge (Eppendorf). The top 200 µl of the solution was removed, and 200 µl of sucrose/glucose solution was mixed with the remaining vesicles, spun, and repeated three times. The density difference between sucrose and glucose allowed for a gentle separation and removal of the excess dye. Evaluation by confocal microscopy was used to verify that the vesicle lumens exhibited a stronger signal and greater concentration of the dextran-488 compared to the exterior solution. Vesicle sizes that are observable by conventional microscopy are limited to 1 µm to 1 mm. Because the images displayed a high contrast between the bilayer labeled with DiD and the background, the number of micelles, SUVs, and LUVs that might interfere with imaging was minimal.

### Fluorescence Resonance Energy Transfer

Recombinant SpTransformer protein, rSpTransformer-E1 (rSpTrf-E1), or BSA was biotinylated with 50 µM of EZ-Link^®^ Sulfo-NHS-LC-LC-Biotin (Thermo Fisher Scientific) following the manufacturer’s instructions and mixed with NeutrAvidin-fluorescein isothiocyanate (NA-FITC) (1:100 dilution; Pierce) according to Lun et al. (12). Biotinylated rSpTrf-E1 labeled with NA-FITC (rSpTrf-E1-FITC) was added to each well of a black 96-well round bottom plate (Corning Costar) containing 100 µl of SUVs. Samples were mixed and immediately excited at 450 nm to initiate FRET and emission was recorded at 560 nm. Excitation was repeated three times for each sample, recorded with a SpectraMax M5 Microplate Reader (Molecular Devices), and analyzed with the microplate data software SoftMax Pro (ver. 5) in the Read Mode setting for Spectrum and Fluorescence. After each reading, additional rSpTrf-E1-FITC was added to the SUVs and FRET was re-evaluated. The concentration of rSpTrf-E1-FITC added to the SUVs ranged from 0 to 10 µg. Background was determined from samples that omitted rSpTrf-E1-FITC and were evaluated for FRET with increasing concentrations of NA-FITC. Negative controls employed 0–10 µg of biotinylated BSA labeled with NA-FITC (BSA-FITC). The background levels were subtracted from the experimental results to generate the net FRET for the SUVs with rSpTrf-E1-FITC and BSA-FITC. A two-tailed, paired *t*-test was used to determine statistical significance among the net FRET results, which was recognized at *p* ≤ 0.05. Means and SDs of the FRET data were calculated for each assay.

### Microscopy

Giant unilamellar vesicles (200–300 µl) in solution were placed in a Granier CELLSTAR^®^ 96-well flat-bottom plate (Sigma-Aldrich) and allowed to settle for 30 min to 1 h and verified by confocal microscopy. Either 10 µM rSpTrf-E1 or 1 µl PBS (background control) was added to a region of the well in which there were many GUVs, which was imaged in multiple fields every 30 s for 30 min. Images were collected using an inverted Zeiss LSM 510 confocal microscope with a 63 × 1.2 NA water objective lens. DiD was excited with HeNe 633 nm laser, and images were collected with an emission range of 650–750 nm. Images with dextran-488 and NBD-PA were collected using an argon 488 nm laser with an emission range of 515–750 nm. Image J (National Institutes of Health^1^) was used to view and assemble the images.

### Circular Dichroism (CD)

Circular dichroism spectra of rSpTrf-E1 were obtained using a measurement range of 190–260 nm with 50 nm/min scanning speed, 1 nm bandwidth, 8 s response time with 1.0 nm data pitch for five scans as described (17). rSpTrf-E1 (0.25 µM) was evaluated alone or in the presence of 1 mM SUVs that were composed of 10% PA:PC or 100% PA after equilibration for at least 10 min and not more than 30 min at rt. Background baseline CD spectra of 10 mM sodium phosphate buffer (pH 7.4) were subtracted from samples including rSpTrf-E1. Boxcar smoothing was used to remove noise from the signal. CD spectra were used to calculate the mean residue ellipticity, or θ, with standard units of degrees (deg) × cm^2^ × dmol^−1^. The fractional helicity was calculated using the ellipticity ratio (*R* = θ_222_/θ_207_) with the spectral data at 222 and 207 nm (25). CD spectra results were deconvoluted to calculate the percentage of protein secondary structure using the CDNN program^2^ (26, 27), and DichroWeb server^3^ (17, 28).

### Vesicle Leakage Assay

Large unilamellar vesicles loaded with ANTS and DPX (see above) were mixed with rSpTrf-E1, and fluorescence was detected with a SpectraMax M5 (Molecular Devices, LLC) in which ANTS was excited at 360 nm and detected at 520 nm. rSpTrf-E1 (10 µM) was added at *t* = 0, and data collection was terminated when the fluorescence signal ceased to increase and appeared to reach a steady state. All analyses were performed with 10 µM lipid concentration and corrected for background fluorescence obtained for the lipids alone. LUVs loaded with ANTS and DPX were lysed with 0.1% Tween-20 and used as the positive control to determine the maximum fluorescence in the absence of quenching. Fractional fluorescence (*f_t_*) was calculated by

$$f_{t}=\left(F_{t}-F_{\text{0}}\right)/\left(F_{\text{max}}-F_{\text{0}}\right),$$

where *F*_0_ is the initial fluorescence measured prior to rSpTrf-E1 addition, *F*_max_ is the maximum fluorescence obtained when loaded LUVs were lysed in detergent, and *F_t_* is the fluorescence measured at time *t*. The kinetics of ANTS leakage was modeled and fitted with a simple three variable equation.

$$f_{t}(t)=A_{0}+A_{1}[1-e^{-k_{1}t}].$$

In this model, *A*_0_ is the fraction that is released initially, and *A*_1_ is the fraction that is released with a rate of *k*_1_ per time (*t*) in seconds. All kinetic curves were fit using Matlab (The Mathworks, Inc.) in which the three variables were varied until the sum of the square error was minimized (29–32).

## Results

### rSpTrf-E1 and Recombinant Fragments Bind to Specific Lipids

Native SpTrf and HeTrf proteins in cells are associated with vesicle membranes and are present on the exterior surface of the plasma membrane (8, 14, 20), which does not agree with predictions from amino acid sequences that these proteins have no obvious means for membrane association. Consequently, to determine whether the membrane association observed by microscopy could be replicated using other approaches, rSpTrf-E1 and the three recombinant fragments (Figure 1A) of the full-length protein were incubated with a lipid-embedded nylon strip to screen for binding to phospholipids, a few phosphatidylinositol (PtdIns) lipids, and seven other biologically important lipids. rSpTrf-E1 and the rGly-rich fragment bound only to PA, whereas the rHis-rich fragment bound to PA and to phosphatidylinositol-4-phosphate [PtdIns(4)P] although the spot intensity for PtdIns(4)P suggested weaker binding (Figure 1B). Alternatively, the rC-Gly fragment bound weakly only to phosphatidylserine (PS). The structures of PA and PtdIns(4)P to which rSpTrf-E1 and the rGly-rich and rHis-rich fragments bound suggested that exposed phosphates with extended or terminal chemical orientations may be the basis for interactions with the proteins. This result was in agreement with speculations that rSpTrf-E1 bound to charged groups including phosphates on LPS (12) and the sulfate group on SDS (17).

### rSpTrf-E1 Interacts Closely with PA

**Figure 2:**
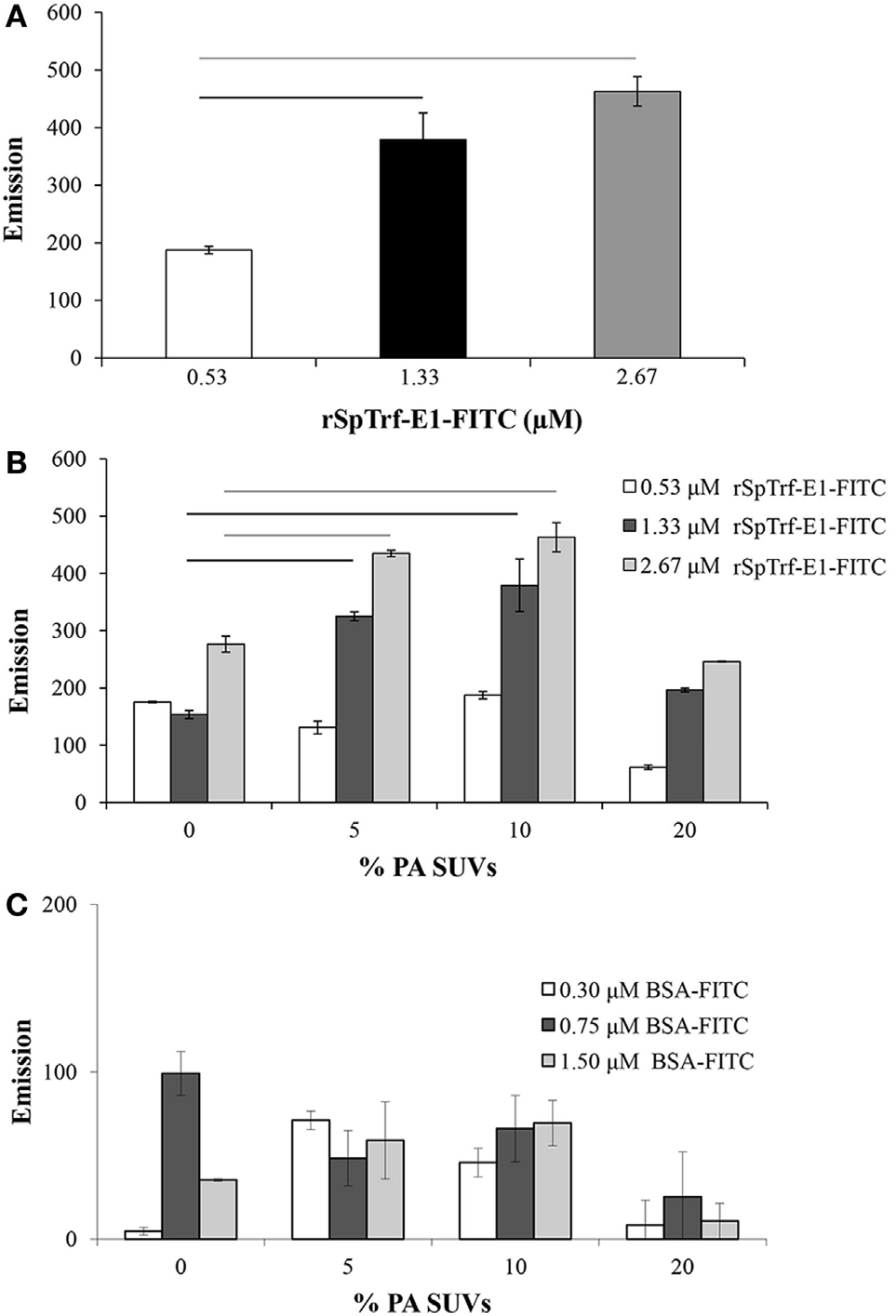
**Small unilamellar vesicles (SUVs) composed of 10% PA:PC with DiI plus recombinant SpTransformer protein, rSpTransformer-E1 (rSpTrf-E1)-FITC generates fluorescence resonance energy transfer (FRET)**. **(A)** FRET increases with increasing concentration of rSpTrf-E1-FITC when mixed with 10% phosphatidic acid (PA):phosphatidylcholine (PC) SUVs. **(B)** FRET evaluation at varying percentages of PA in PC liposomes shows that 10% PA:PC SUVs exhibit optimal FRET when mixed with either 1.33 or 2.67 µM rSpTrf-E1-FITC (not statistically different). Reduced FRET for SUVs with 20% PA:PC may have been the result of self-quenching, and these data are not included in the statistical analysis. Bars represent mean and SE, and black and gray horizontal lines indicate statistical significance at *p* < 0.05. **(C)** SUVs composed of PC plus indicated percentages of PA show low levels of FRET in the presence of BSA-FITC. These levels are not different from SUVs in the presence of NA-FITC and are significantly lower than FRET with SUVs in the presence of all concentrations of rSpTrf-E1-FITC. Horizontal bars indicate significance at *p* < 0.05. FRET was initiated with excitation at 450 nm, and emission was recorded at 560 nm.

To verify a close interaction between rSpTrf-E1 and PA, FRET was used to evaluate the emission from FITC linked to rSpTrf-E1 to excite DiI in liposome membranes containing PA. The recombinant fragments were not evaluated in the experiments using FRET because the rGly-rich and rHis-rich fragments gave the same results as rSpTrf-E1 on the lipid strips, and the rC-Gly fragment, which multimerizes upon isolation (12), resulted in a different lipid-binding signature that may not reflect the activities of the intact protein. PC was used as the neutral lipid background to stabilize the negatively charged PA because it is commonly found in most cell membranes (33) and was not bound by rSpTrf-E1 (Figure 1B). In an initial experiment, SUVs composed of 10% PA:PC and labeled with DiI were mixed with increasing concentrations of rSpTrf-E1-FITC and energy transfer was measured at 560 nm. Background was determined by the 560 nm emission of 10% PA:PC SUVs and DiI plus increasing concentrations of unlabeled rSpTrf-E1, which was subtracted from the experimental signal to determine the net FRET. FRET results, which are generally accepted to indicate that molecules are within 10 nm of each other, suggested that FITC and DiI, and therefore rSpTrf-E1-FITC and PA, were in very close association (Figure 2A). To determine the optimal percentage of PA in liposomes to optimize FRET with rSpTrf-E1-FITC, SUVs with increasing concentrations of PA were compared to SUVs composed only of PC (background control). In general, net FRET increased with increasing concentrations of rSpTrf-E1-FITC plus SUVs with a given percentage of PA, and SUVs composed of 5–10% PA:PC produced significantly increased FRET with increasing concentrations of rSpTrf-E1 (Figure 2B). FRET resulting from 0.53 µM rSpTrf-E1-FITC did not change with respect to the percentage of PA in the SUVs suggesting that this concentration of rSpTrf-E1-FITC was too low to initiate FRET. SUVs composed of 20% PA:PC showed signs of self-quenching and produced lower net FRET (Figure 2B) likely because the higher concentration of NA-FITC in the controls interfered with emission detection (34). Therefore, results with 20% PA:PC SUVs were not included in the statistical analyses and were not evaluated further. rSpTrf-E1-FITC binding to SUVs containing PA appeared to be specific, because increasing concentrations of BSA-FITC did not show significant changes in net FRET when evaluated with SUVs with various percentages of PA (Figure 2C). FRET emission results suggested that rSpTrf-E1 associated closely with PA (Figure 2B) and confirmed results for rSpTrf-E1 binding to the lipid-embedded nylon strip (Figure 1B). The combination of 10% PA:PC liposomes and 2.67 µM rSpTrf-E1-FITC was used for further analyses.

### rSpTrf-E1 Causes Budding, Invagination, Fusion, and Leakage of GUVs

To visualize the close physical association between rSpTrf-E1 and liposomes containing PA suggested by FRET, rSpTrf-E1-FITC was added slowly to one edge of a well in a flat-bottom plate containing GUVs labeled with DiD. Images captured by confocal microscopy over 20 min (four scans per min) did not show a colocalization of FITC and DiD likely because of limited sensitivity by the imaging system, which did not detect the low concentration of rSpTrf-E1-FITC relative to DiD. However, unexpected morphological changes to the GUVs were observed in the presence of rSpTrf-E1-FITC, which were not observed in the absence of the protein. After about 9 min, GUVs showed evidence of budding, fusion, and invagination (Figure S1 and Movie S1 in Supplementary Material; white arrows indicate fusion and budding). When the GUVs were imaged again after a several hours, they were completely lysed.

**Figure 3:**
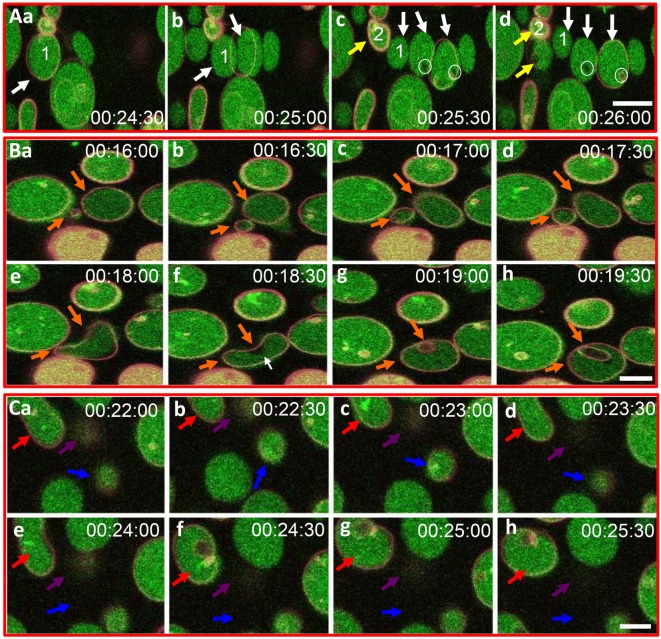
**Recombinant SpTransformer protein, rSpTransformer-E1 (rSpTrf-E1), induces giant unilamellar vesicles (GUVs) to bud, fuse, invaginate, leak, and disappear**. **(A)** Confocal microscopy images show budding of two independent GUVs into two or three smaller vesicles (a–d, white and yellow arrows). Leakage of dextran-488 appears as black spaces in the lumen of two GUVs (c,d, white circles). **(B)** Images show GUV fusion between two GUVs (a–e, orange arrows), leakage at the convex curve of the membrane (white arrow), which is the site of invagination of the fused GUV (f–h, orange arrows). **(C)** Images show invagination (a–h, red arrows), lysis (a–h, blue arrows), and a slow decrease in dextran-488 fluorescence in a GUV (a–h, purple arrows) suggestive of slow leakage leading to lysis. Image acquisition is every 30 s as indicated after the addition of rSpTrf-E1. All scale bars indicate 10 μm.

Based on the initial results suggesting that rSpTrf-E1 induced morphological changes in GUVs, improved visualization of GUVs employed dextran-488 in the lumen, DiD in the membrane, and unlabeled rSpTrf-E1, with images captured every 30 s for 20–40 min by confocal microscopy. Images confirmed the initial results and showed changes in membrane curvature for some GUVs after the addition of rSpTrf-E1 that appeared as budding, invagination, and perhaps lysis (Figure 3). Similar morphological changes were not observed for GUVs in the absence of rSpTrf-E1, which remained as spheres for the duration of the observations (Figure S2 in Supplementary Material). A progression of budding for two GUVs over 2.5 min resulted in the appearance of two or three smaller sized GUVs (Figure 3A, a–d, white and yellow arrows). GUV fusion was also observed in which two different sized GUVs came together and fused forming a kidney bean-shaped GUV (Figure 3B, a–e; orange arrows). This kidney bean-shaped GUV proceeded to invaginate into a multilamellar vesicle (Figure 3B, f–h) with an internal vesicle labeled with DiD but without dextran-488 in the lumen (Figure 3B, g,h; orange arrows). GUV invagination was also observed in which an elongated vesicle changed its morphology to a multilamellar GUV over 3 min in which the resulting internal vesicle was also devoid of dextran-488 in the lumen (Figure 3C, a–h; red arrows). What appeared to be GUV lysis in the presence of rSpTrf-E1 was observed when a bright green fluorescent GUV disappeared within 30 s (Figure 3C, d,e; blue arrows) suggesting that the protein may induce membrane destabilization leading to the complete release of vesicle contents.

### rSpTrf-E1 Causes Leakage of Luminal Contents from LUVs

**Figure 4:**
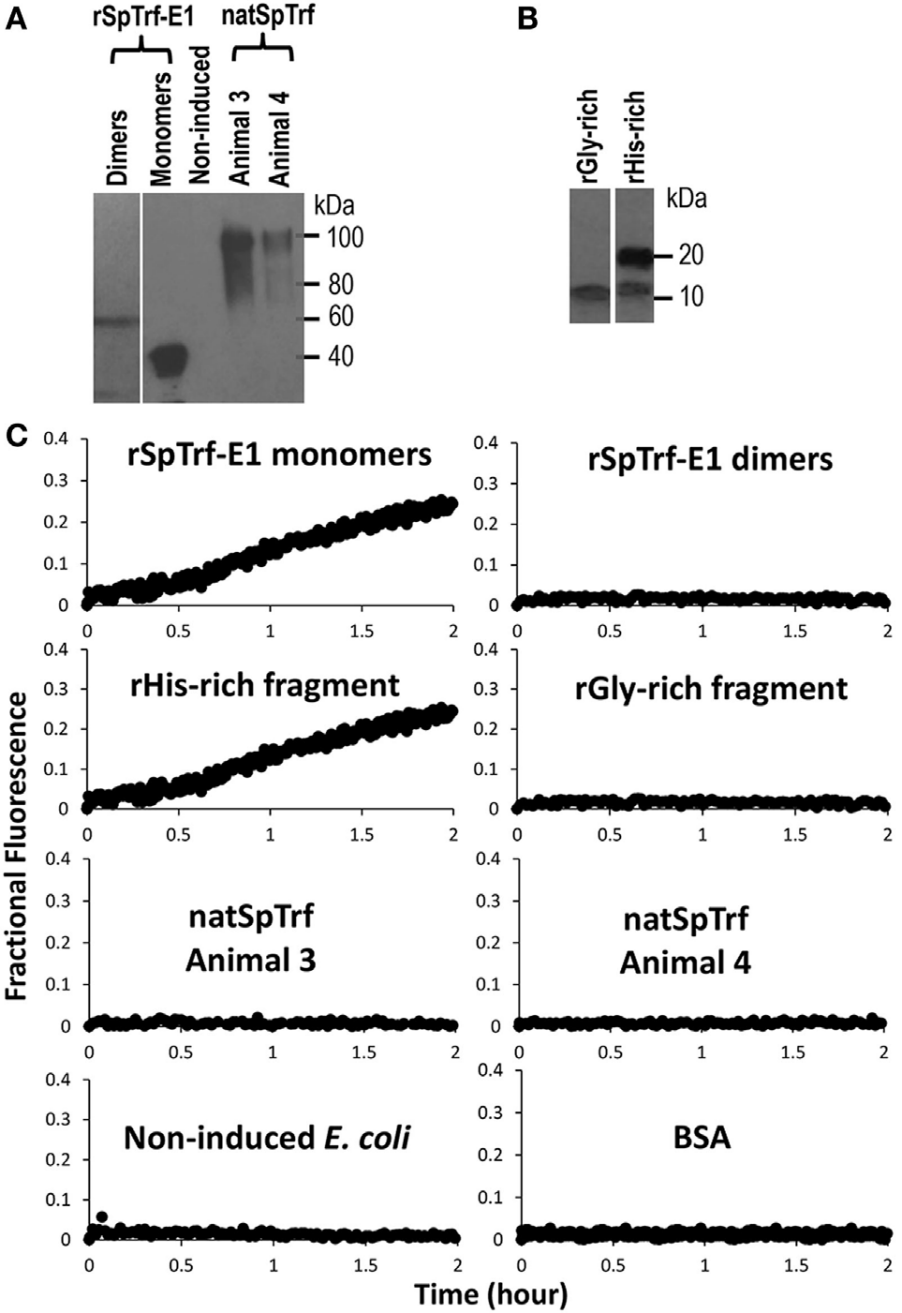
**Recombinant SpTransformer protein, rSpTransformer-E1 (rSpTrf-E1), monomers and the rHis-rich fragment induce luminal content leakage from the large unilamellar vesicles (LUVs)**. **(A)** A Western blot evaluated with anti-SpTrf antisera shows dimers and monomers of rSpTrf-E1, unknown proteins from non-induced bacteria (Non-induced) that were processed following the same sample preparation as the recombinant proteins, and natSpTrf proteins isolated from two sea urchins by nickel affinity according to Sherman et al. (16). **(B)** A Western blot evaluated with anti-SpTrf antisera shows the rGly-rich and rHis-rich fragments expressed in *E. coli* and isolated by nickel affinity. The rHis-rich fragment shows partial degradation from 20 to 15 kDa as reported previously (12). **(C)** LUVs incubated with rSpTrf-E1 monomers and the rHis-rich fragment (both at 10 µM) induce fluorescent dye leakage. The other protein isolates are not active.

In addition to the apparent GUV invagination, fusion, budding, and lysis events, an uneven distribution of the green dextran appeared as dark regions within the lumen of some GUVs (Figure 3A, c,d; white circles) suggesting that the dextran-488 may have leaked from the GUVs. For example, a dark region in the lumen was noted near the convex curve in the fused GUV (Figure 3B, f; white arrow) just prior to invagination that occurred at this same location. This change in the distribution of luminal dextran-488 was not observed in the control GUVs in the absence of rSpTrf-E1 (Figure S2 in Supplementary Material). Although, lysis of one particular GUV was suggested above (Figure 3C, a–e; purple arrows) an alternative possibility was that rSpTrf-E1 may alter the membrane to allow dextran solution to escape from the liposome and diffuse into the surrounding buffer to concentrations below detection by microscopy. To verify that these changes were due to lysis and/or leakage and to quantify the leakage rate, LUVs were loaded with ANTS (fluorescent dye) and DPX (quencher) and incubated with rSpTrf-E1 (both monomers and dimers) and with Ni-natSpTrf proteins isolated from two different sea urchins (Figure 4A). Based on the identification of PA binding (Figure 1), both the rGly-rich and rHis-rich fragments (Figure 4B) were also evaluated for GUV leakage. The rC-Gly fragment was not employed in this assay because it multimerizes upon isolation, bound poorly to PS and did not bind to PA (Figure 1B), and shows non-specific binding to a range of foreign targets (12). Negative control proteins included BSA and unknown proteins isolated by nickel affinity from non-induced *E. coli* that served as the negative control for the isolation protocol for the recombinant proteins (Figure 4A). After 2 h, increased ANTS fluorescence was only detected from LUVs incubated with monomeric rSpTrf-E1 or the rHis-rich fragment, which could be measured based on the separation of ANTS from DPX upon release from the liposome and diffusion into the buffer (Figure 4C). Although the rGly-rich fragment bound to PA (Figure 1B), it did not induce luminal content leakage from the LUVs. Leakage was not induced by either dimerized rSpTrf-E1 or the Ni-natSpTrf protein isolates, which were entirely multimerized upon collection from two sea urchins. This was the first evidence that dimerized rSpTrf-E1 was not active compared to the monomer and inferred that multimerization of the natSpTrf proteins may have been an attribute of the lack of leakage activity. These results also suggested differences in the activities of the His-rich and Gly-rich regions in rSpTrf-E1.

**Figure 5:**
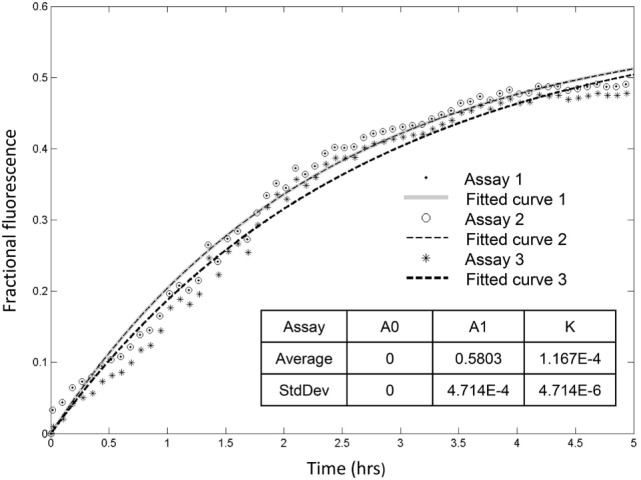
**Recombinant SpTransformer protein, rSpTransformer-E1 (rSpTrf-E1), induces leakage that plateaus at about 5 h**. Three independent leakage assays with 10 µM rSpTrf-E1 show that reaching the fluorescence leakage plateau requires about 5 h. The table insert shows that the results are reproducible at 0 initial leakage rate (*A*_0_) when rSpTrf-E1 is added to the sample with average fraction of ~0.58 (*A*_1_) that is released with an average kinetic rate (*k*) of ~1.17 × 10^−4^ s^−1^.

Recorded fractional fluorescence for ANTS release in the presence of rSpTrf-E1 or the rHis-rich fragment did not plateau by 2 h (Figure 4) indicating that neither protein had induced maximum leakage within that time frame. Because rSpTrf-E1 and the rHis-rich fragment had very similar fractional fluorescence results, only rSpTrf-E1 was used in the subsequent leakage assay of 5 h to identify the maximum leakage by reaching the fluorescence plateau. Three independent assays demonstrated that rSpTrf-E1 induced reproducible leakage, and these results were well described by a two-step process of an instantaneous first step and a slower rate-determining second step with a measurable rate (Figure 5). Calculations yielded an average leakage fraction of ~0.58 (*A*_1_) that was released with an average kinetic rate (*k*_1_) of ~1.17 × 10^−4^ s^−1^. The fractional fluorescence showed a slow leakage process for 10 µM rSpTrf-E1 that required 4–5 h before the fluorescence reached a stable plateau. The mode of action for rSpTrf-E1 appeared to require more time and may be more subtle and less drastic than interactions between known antimicrobial peptides and membranes (35). Although the actual sequence of events of natSpTrf proteins binding to targets *in vivo* is unknown and may have several steps and involve multiple natSpTrf isoforms, the kinetic findings for rSpTrf-E1 suggested a general interpretation of a first step as the protein binding to PA, and a second step as a specific interaction or re-arrangement of proteins and lipids that led to membrane destabilization and leakage.

### rSpTrf-E1 Causes PA to Cluster

**Figure 6:**
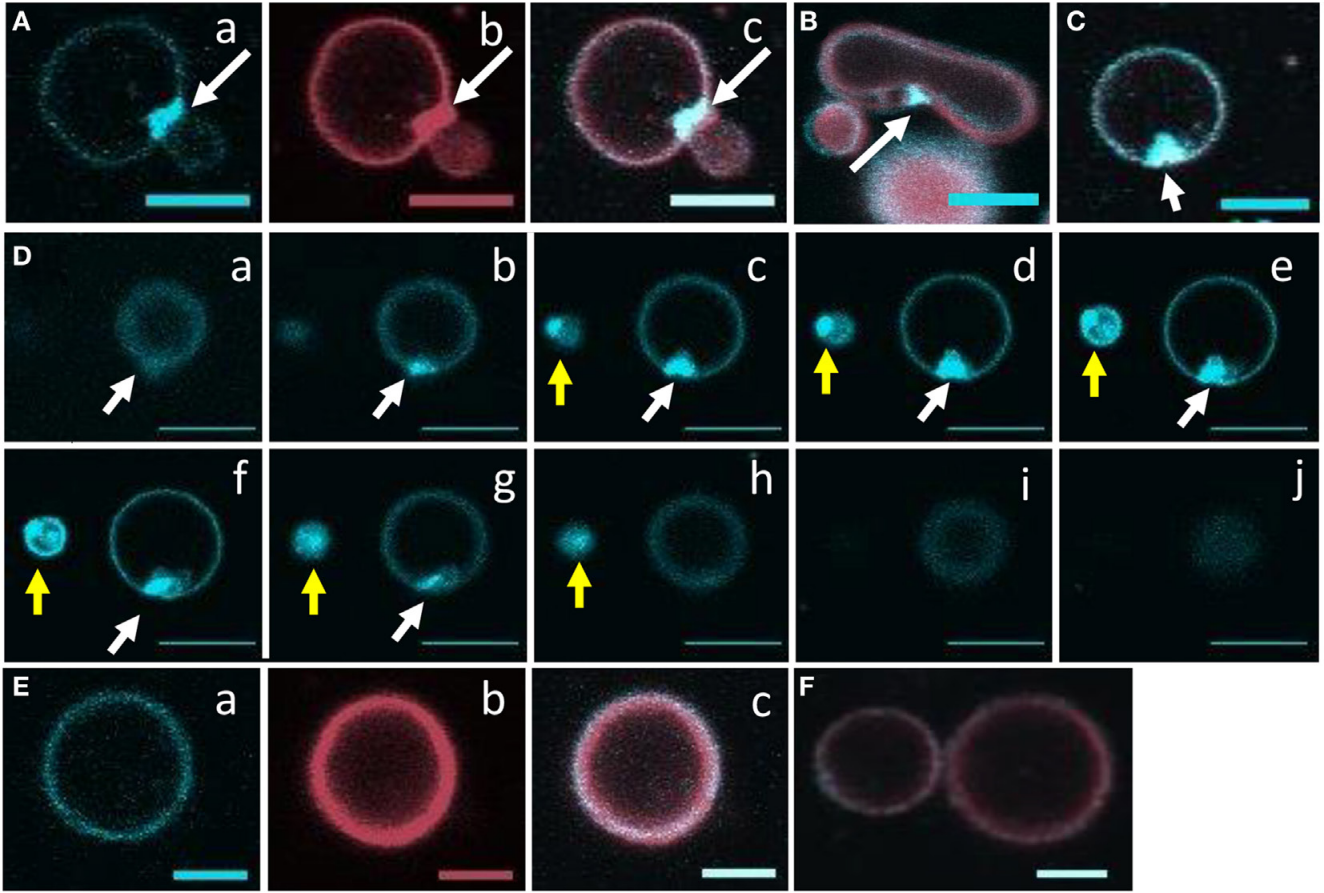
**Recombinant SpTransformer protein, rSpTransformer-E1 (rSpTrf-E1), causes NBD-PA to cluster in the lipid bilayer**. Confocal microscopy images were captured 20 min after the addition of rSpTrf-E1 to giant unilamellar vesicles (GUVs) that are composed of 6% NBD-PA, 4% PA, and 90% PC (100% g/ml). **(A)** An NBD-PA cluster (arrow) is present at the intersection of two GUVs. Images show NBD-PA (a), DiD in the GUV membrane (b), and the merge (c). **(B)** The merged image shows an NBD-PA cluster (arrow) at a region of concave curvature of a GUV membrane. **(C)** A single cluster of NBD-PA is present in a GUV membrane. **(D)** A *Z*-stack of images (a–j) from the bottom to the top of two GUVs (white and yellow arrows) shows that each GUV has a single NBD-PA cluster. **(E)** A GUV without added rSpTrf-E1 shows no change in NBD-PA distribution at 20 min. Images NBD-PA (a), DiD in the GUV membrane (b), and the merge (c). **(F)** Two GUVs without added rSpTrf-E1 show an even distribution of NBD-PA at 20 min. All scale bars indicate 10 μm.

**Figure 7:**
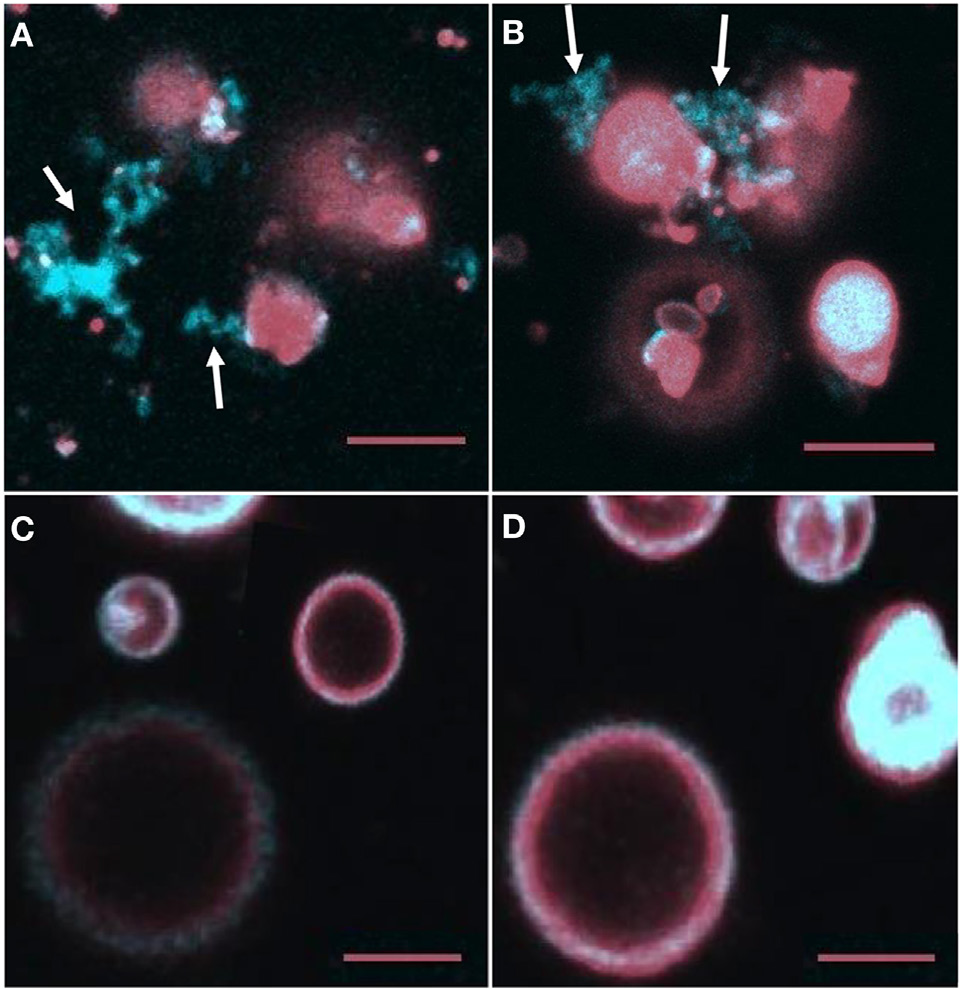
**NBD-PA becomes separated from giant unilamellar vesicles (GUVs) after 2 h of incubation with recombinant SpTransformer protein, rSpTransformer-E1 (rSpTrf-E1)**. **(A,B)** NBD-PA (arrows) forms clusters that are separated from the GUVs after 2 h of incubation with rSpTrf-E1. **(C,D)** GUVs in the absence of rSpTrf-E1 show an even distribution of NBD-PA and DiD at 2 h. Differences in the GUV sizes and content of NBD-PA are an outcome of GUV preparation. All images are merged for NBD-PA (blue) and DiD (red) as captured by confocal microscopy. All scale bars indicate 10 μm.

Giant unilamellar vesicles in the presence of rSpTrf-E1 showed changes in membrane curvature leading to invagination or budding, which was not observed when PA was not incorporated into the GUVs. To determine whether changes in membrane curvature was a result of PA clustering, which might occur because PA has a conical shape resulting from the very small phosphate head group (36), GUVs of 6% NBD-PA, 4% PA, 90% PC plus DiD were imaged in the presence or absence of rSpTrf-E1. The 20-min time point was chosen to begin imaging because this was the point at which most changes in morphology were observed for GUVs loaded with dextran-488 after the addition of rSpTrf-E1 (Figure 3). Images of selected GUVs after the addition of rSpTrf-E1 showed clustered NBD-PA that formed bright blue fluorescent patches in the lipid bilayer (Figures 6A–D, white arrows; Figures S3A–C in Supplementary Material). NBD-PA clusters were sometimes present at the intersection of two GUVs (Figure 6A), in regions of membrane curvature (Figure 6B), and positioned at points of contact between membranes within multilamellar GUVs (Figure S3B in Supplementary Material). Confocal *Z*-stack images of the NBD-PA clusters in GUVs in the presence of rSpTrf-E1 showed that a single PA cluster was typically present per liposome rather than multiple clusters (Figure 6D). These morphological attributes were consistent with PA clusters being the basis for membrane curvature, budding, and invagination. NBD-PA clustering in GUVs was not observed in the absence of rSpTrf-E1 and showed an even distribution in the spherical GUVs (Figures 6E,F; Figures S3D–G in Supplementary Material). After 2 h of incubation of GUVs with rSpTrf-E1, the NBD-PA appeared in disordered clusters associated with but outside of the GUV membranes (Figures 7A,B; Figures S3H,I in Supplementary Material). In the absence of rSpTrf-E1 at 2 h, there was an even distribution of NBD-PA in the GUVs and no clusters of NBD-PA appeared within or outside of the GUV membranes (Figures 7C,D). These results suggested that the clustering of PA induced by rSpTrf-E1 proceeded to PA extraction from the membranes.

Many of the vesicles in the experiments reported here displayed no changes in membrane morphology in the presence of rSpTrf-E1 (Figure 3), and several factors may have been the basis for this observation. First, confocal imaging only has a small window for observation and recording of the events, which limited the number of vesicles that could be evaluated. Second, the addition of rSpTrf-E1 was added to an edge of the wells to minimize disturbing the settled vesicles, and likely induced a gradient of the protein across the well as it diffused into the solution. Third, variations in the PA concentration among vesicles are known to occur (see Figures S3D–G in Supplementary Material). It was likely that a combination of all resulted in variations in the numbers of PA–rSpTrf-E1 interactions among individual vesicles that led to morphological changes in some vesicles and not in others.

### rSpTrf-E1 Transforms from Disordered to α Helical in the Presence of PA and PA/PC Liposomes

**Figure 8:**
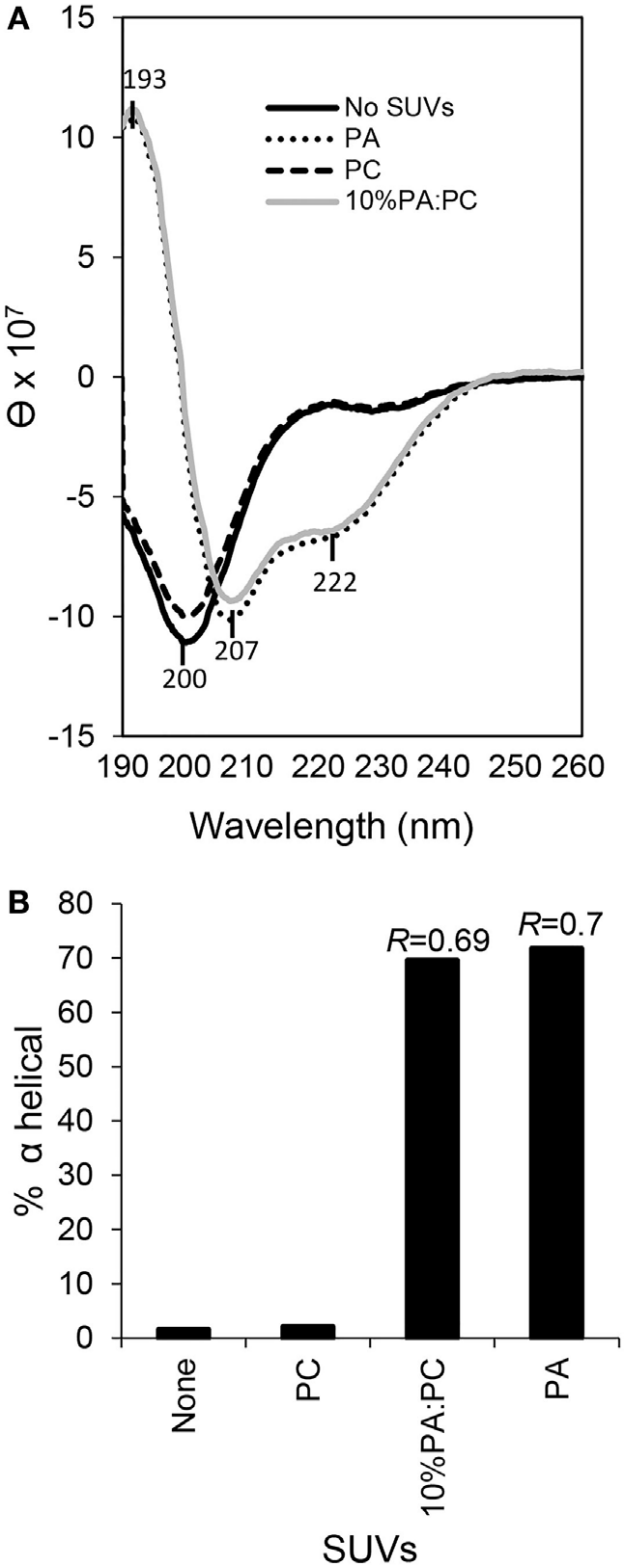
**Secondary structure of recombinant SpTransformer protein, rSpTransformer-E1 (rSpTrf-E1), transforms from intrinsically disordered to α helical in the presence of phosphatidic acid (PA) small unilamellar vesicles (SUVs)**. **(A)** CD spectra show intrinsic disorder or random coils for 0.25 mM rSpTrf-E1 in 10 mM sodium phosphate buffer in the absence of PA or in the presence of 100% phosphatidylcholine SUVs (PC). rSpTrf-E1 transforms to α helical secondary structure in the presence of 10% PA:PC SUVs or 100% PA SUVs (PA). θ is the mean residue ellipticity with standard units of degrees × cm^2^ × dmol^−1^ as described (17). **(B)** The percentage of α helical structure for rSpTrf-E1 is 1.57% in the absence of lipids and 2.1% in the presence of PC. However, in the presence of 100% PA SUVs or 10% PA/PC SUVs, the α helical structure of rSpTrf-E1 is 69.6 and 71.8%, respectively. The percentage of secondary structure for rSpTrf-E1 in the presence of PA are based the deconvolution of the spectra data using the DichroWeb server. The *R* values [ellipticity ratios: *R* = θ_222_/θ_207_ shown in panel **(A)**] are indicated for the CD analysis of rSpTrf-E1 with 10% PA:PC and for 100% PA (17).

Previous bioinformatic predictions and CD analysis of rSpTrf-E1 indicated that it is an IDP that transforms from disordered to α helical upon interactions with SDS, TFE, or LPS (12, 17). Based on the structural similarity between SDS (a single acyl chain linked to a sulfate group) and PA (two acyl chains linked to a phosphate head group), we hypothesized that rSpTrf-E1 binding to PA might drive similar secondary structural changes in the protein. Results from CD spectra of rSpTrf-E1 in the presence of PA, either as 100% PA SUVs or as 10% PA:PC SUVs, demonstrated that rSpTrf-E1 transformed from disordered to ~70% α helical structure (Figure 8). In the presence of fully neutral SUVs composed of 100% PC or in the absence of lipids, rSpTrf-E1 remained intrinsically disordered (~2% α helical) in agreement with a disordered structure in the absence of binding targets (17). These results suggested that the interaction between PA and rSpTrf-E1 was similar to observations with SDS and transformed the protein to α helical secondary structure.

## Discussion

Native SpTrf and HeTrf proteins are found in all morphotypes of sea urchin phagocytes, on the surface of small phagocytes in association with the plasma membrane and with the membranes of cytoplasmic vesicles (14, 19–21). But rather than integrated into membranes *via* transmembrane regions or associated through GPI linkages, rSpTrf-E1 and its rGly-rich and rHis-rich fragments may associate with membranes, at least in part, by binding directly to PA or other lipids with exposed phosphate groups. Interactions between rSpTrf-E1 and liposomes that include PA alter membrane curvature, which has been noted as a characteristic of cone-shaped PA in other systems (36, 37), and correlates with PA clustering that is likely the basis for budding, fusion, and invagination. Both monomeric rSpTrf-E1 and the rHis-rich fragment cause slow leakage of luminal contents demonstrating that the proteins do not induce sudden membrane disruption unlike activities of some antimicrobial peptides (35, 38). Both the rGly-rich and rHis-rich fragments bind PA suggesting that the full-length protein is at least bivalent, which is similar to results from other proteins with PA-binding domains that are likely multivalent (39). Although the rGly-rich fragment binds to PA, it does not induce leakage indicating that the His-rich region of rSpTrf-E1 is likely responsible for this activity. When rSpTrf-E1 and Ni-natSpTrf proteins are dimerized or multimerized prior to mixing with liposomes they do not induce leakage suggesting that only monomers are active. Irreversible multimerization among natSpTrf proteins has been noted repeatedly (12, 14–16), and we speculate that this may be an intrinsic control mechanism for natSpTrf proteins that do not bind quickly to pathogens or to other non-self targets and may limit the potential for destructive activities toward self. In the presence of PA and SDS, which have similar anionic and amphipathic structures, rSpTrf-E1 transforms from disordered to α helical structure (17). Similarly, speculations on the PA-binding domains from yeast SNARE proteins also suggest protein disorder in the cytosol that alters to amphipathic α helical structure after binding to PA (39). Our findings provide the first evidence of a possible means by which natSpTrf proteins may associate with exposed phosphate groups on PAMPs including PA in membranes and that the His-rich region within rSpTrf-E1 has destabilizing activities for simple membranes.

### Binding between rSpTrf-E1 and PA

There is no commonly recognized site or domain for any protein that binds PA; however, clusters of positively charged amino acids are speculated to be responsible for this interaction (40). The amino acid composition of rSpTrf-E1 is 24.8% positively charged amino acids [76 of 307 amino acids (aa); 27 His, 4 Lys, 45 Arg; see Table S3 in Ref. (12)]. Similarly, positively charged amino acids compose 30.4% of the rHis-rich fragment (56 of 184 aa; 27 His, 3 Lys, 26 Arg) and 17.5% of the rGly-rich fragment (15 of 86 aa; 1 Lys, 14 Arg), and each of these recombinant proteins binds to PA. In comparison, none of the recombinant proteins tested here bind to diacylglycerol, which is identical to PA but without the phosphate head group, suggesting that the interaction is focused on the phosphate. In addition to PA, the rHis-rich fragment binds to PtdIns(4)P, which also has an exposed phosphate on the inositol head group, although binding appears to have lower affinity compared to PA (Figure 1B). Binding to PtdIns(4)P may require a higher percentage of positively charged amino acids that are present in the rHis-rich fragment compared to the other recombinant proteins tested in this study and may offset the possibility that the phosphate on PtdIns(4)P may be less accessible than on PA. It is noteworthy that rSpTrf-E1 that includes the His-rich region does not bind to PtdIns(4)P suggesting an interaction between the Gly-rich and His-rich regions within the full-length protein to enhance or restrict binding to PA, which is relaxed when the rHis-rich fragment is expressed alone. Although the rC-Gly fragment has 15.4% positively charged amino acids (6 Arg of 39 aa), it does not bind to PA, which may be due to the spacing of the 6 arginines that are spread out as 2 singles and 2 doubles in this short fragment. The lipid binding by the rC-Gly fragment to PS, albeit weak based on spot intensity on the lipid-embedded strip (Figure 1B), may be an example of its characteristic of multimerization upon expression and its expanded range of microbial species to which it binds compared to rSpTrf-E1 (12).

**Figure 9:**
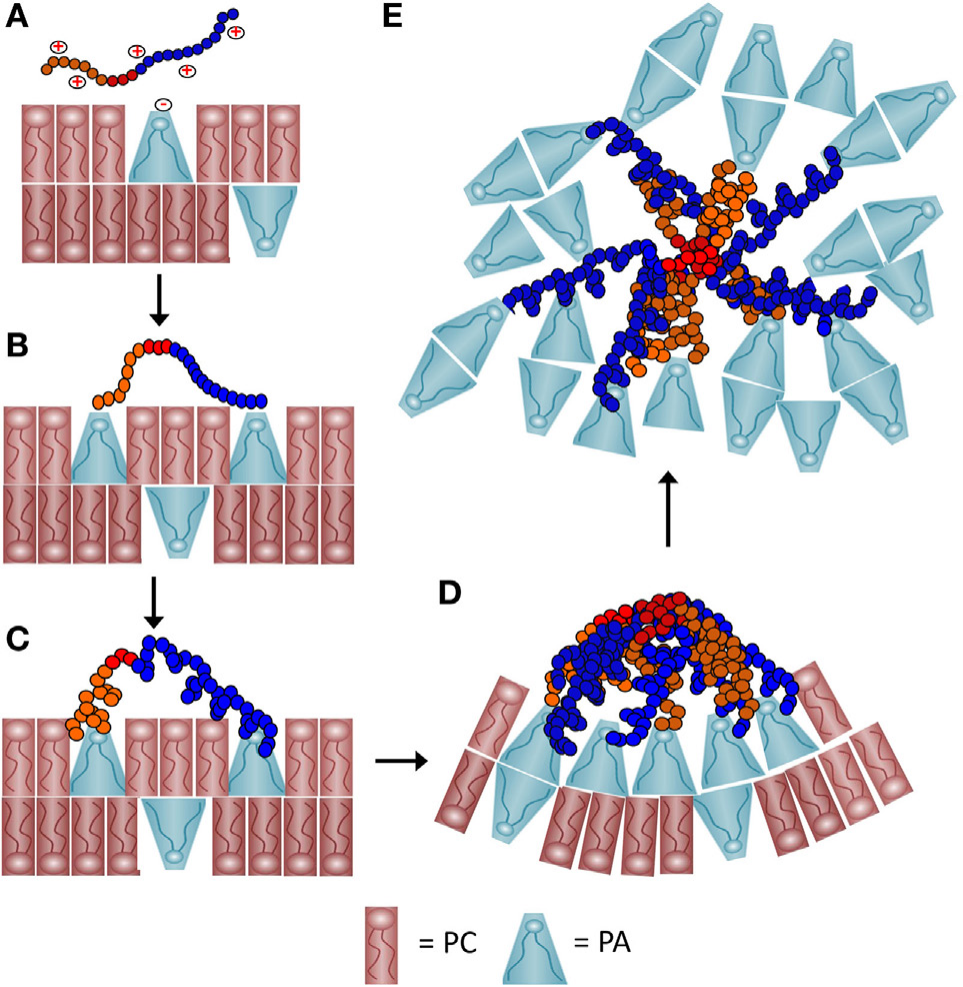
**A schematic representation of a proposed process of how recombinant SpTransformer protein, rSpTransformer-E1 (rSpTrf-E1), may cause phosphatidic acid (PA) clustering and PA extraction from liposomes**. **(A)** The positively charged amino acids (red+) in the Gly-rich region (orange) and the His-rich region (blue) of rSpTrf-E1 interact with the negatively charged (red−) phosphate head group of PA (blue cone-shaped lipid) through initial electrostatic attractions. Phosphatidylcholine (PC) (red rectangular lipid) is 90% of the lipids in the liposomes. **(B)** The positively charged amino acids from both the Gly-rich and His-rich regions of rSpTrf-E1 each bind to the phosphate head group on PA. The C terminal region of the Gly-rich (C-Gly) region (red) does not bind to PA. **(C)** Binding between rSpTrf-E1 and PA causes the protein to undergo a structural transformation from disordered to α helical. **(D)** The C-Gly region of α helical rSpTrf-E1 interacts with other C-Gly regions in other rSpTrf-E1 proteins causing protein multimerization or aggregation that brings PA into clusters. Clusters of cone-shaped PA induce liposome membrane curvature that leads to budding, invagination and fusion (not shown). **(E)** The C-Gly region continues to multimerize rSpTrf-E1 proteins into larger aggregates that extract PA from the liposomes and result in disordered PA clusters that are separated from the liposomes.

The relatively high content of positively charged amino acids in rSpTrf-E1 and the rHis-rich and rGly-rich fragments are congruent with a proposed molecular model of an electrostatic/hydrogen bond switch (40) that may explain the interactions between the monomeric rSpTrf-E1 and PA in a lipid bilayer. This model proposes that upon the initial attraction, the positively charged amino acid side groups in the protein may interact electrostatically with PA in the bilayer and form hydrogen bonds with the negatively charged and exposed phosphate. When in close proximity, the hydrogen bonds between the negative charges on the phosphates and positively charged side groups increase due to deprotonation that strengthens the electrostatic attraction (40). The enhanced negative charges plus hydrogen bonds may result in a tight bond between PA and rSpTrf-E1 or the recombinant fragments resulting in docking of the protein to the lipid (Figures 9A,B). Speculations on the electrostatic interactions between rSpTrf-E1 and phosphate groups are consistent with the previous report demonstrating that rSpTrf-E1 binds to LPS (12). Anionic phosphates on LPS are present on the glucosamine disaccharide in lipid A and also on the polysaccharide core (41) and these phosphates may also form charge-based electrostatic interactions with the positively charged amino acids in rSpTrf-E1.

### rSpTrf Interactions with Liposome Membranes Containing PA

#### rSpTrf-E1 Clusters PA in Liposome Membranes

Recombinant SpTransformer protein, rSpTransformer-E1 (rSpTrf-E1), causes PA to cluster in liposome membranes as observed by the changes in the distribution of NBD-PA. This suggests that rSpTrf-E1 is bivalent, binds two PA molecules, and once bound through electrostatic interactions, it transforms from disordered to α helical structure (Figures 9A–C). This structural change may lead to or be concurrent with multimerization among rSpTrf-E1 proteins bound to PA on the lipid membrane that would bring PA into visible clusters (Figure 9D). The interaction time required for changes to become evident would depend on the number of rSpTrf-E1 proteins bound to PA on a particular liposome, the number of PA molecules in that membrane, and the fluidity of the membrane. Based on the conical shape of PA, its enrichment into clusters would be expected to promote membrane curvature (Figure 9D) (42) leading to the morphological changes observed as budding, fusion, and invagination. Membrane curvature reported here for liposomes is in agreement with PA involvement in membrane curvature in cells including (i) mitochondrial fusion and fission (43, 44); (ii) vesicle formation by generating membrane curvature in the Golgi complex (45), and (iii) membrane dynamics and vesicle trafficking along the secretory pathway including membrane fusion and exocytosis (46–48). The level of PA is elevated in vertebrate macrophages upon activation and functions in signal transduction to induce endocytosis, fusion of perinuclear vesicles with the plasma membrane, and immune activation of these cells (49). Enrichment of cone-shaped PA at sites of closely apposed membranes facilitates fusion to complete the formation of phagosomes, endosomal vesicles, and the process of exocytosis. Our observations of PA clustering induced by rSpTrf-E1, the positions of those clusters at regions of membrane curvature and at intersections of contact between two liposomes are consistent with the activities of PA in intact cells.

#### rSpTrf-E1 Extracts PA from Liposome Membranes

The second level of interactions between rSpTrf-E1 and liposomes containing PA is the apparent extraction of PA from the membranes after 2 h of incubation. This phenomenon may be the outcome of the Gly-rich and His-rich regions of rSpTrf-E1 each binding to a PA molecule followed by the transformation of the protein to α helical, the diffusion along the membrane of rSpTrf-E1–2PA complexes into close association with each other, and the multimerization of the proteins into larger complexes that is mediated by the rC-Gly region (Figure 9D). This would initially appear as large clusters of PA followed by continued multimerization of rSpTrf-E1-2PA not only within but between liposomes to generate complexes large enough to extract PA from liposome membranes (Figure 9E). This would require overcoming the PA acyl chain associations within the membrane and their extraction into the aqueous buffer, after which the PA acyl chains would likely associate with each other. A possible transition from membrane clusters of PA to extracted clusters is consistent with the image in Figure S3C in Supplementary Material. The final outcome of this process is disorganized clusters of PA that are distinct from the residual liposomes (Figure 7; Figure S3 in Supplementary Material).

### rSpTrf-E1 Causes Liposome Leakage

The change in distribution of PA in membranes after mixing with rSpTrf-E1 correlates with both the appearance of dark regions within lumens of liposomes loaded with dextran-488 and the slow leakage of luminal contents. The change in liposome membrane permeability requires a 20-min interaction time with rSpTrf-E1 or the rHis-rich fragment before leakage becomes evident. Although the rGly-rich fragment binds to PA, it does not induce leakage, indicating that the rHis-rich fragment and the His-rich region of rSpTrf-E1 are responsible for altering the characteristics of the liposome membrane to induce leakage. Leakage by the rHis-rich fragment also indicates that protein multimerization after PA binding is not required for the process because the rC-Gly region, which drives multimerization (12), is not included in the rHis-rich fragment (Figure 1A). This suggests that rSpTrf-E1 may have two activities that alter liposomes: (i) those that lead to membrane destabilization and leakage and (ii) those that lead to membrane curvature and changes in liposome morphology. However, these two activities may occur simultaneously in which PA binding leads to (i) membrane destabilization and eventual luminal leakage and (ii) PA clustering that leads to membrane curvature and PA extraction. For example, apparent luminal leakage associated with membrane curvature followed by invagination is illustrated for the bean-shaped GUV in Figure 3B, f–h (white arrow). It is not known whether membrane destabilization and leakage observed for liposomes in the presence of rSpTrf-E1 has an equivalent *in vivo* for natSpTrf activity.

## Conclusion

We report that rSpTrf-E1 associates with the phospholipids PA and PtdIns(4)P. Although these results suggest a means by which this recombinant protein may associate with sea urchin coelomocytes and/or bacterial membranes, it is not known whether PA binding is an important interaction between natSpTrf proteins and membranes of intact cells. This is because there is no information on the phospholipid composition of sea urchin coelomocytes or the marine bacteria, *V. diazotrophicus*, to which rSpTrf-E1 and natSpTrf proteins are known to bind (12). PA is present in small quantities in most internal cellular membranes and is critical for many physiological functions including (i) serving as the precursor for phospholipid synthesis, (ii) involvement in important stress signaling pathways in plants and animals, and (iii) activities in enzyme activation, protein recruitment, cell stress response, and cell signaling (37, 47, 50–52). PA is elevated on the cytoplasmic side of the plasma membrane in vertebrate phagocytes (49) particularly during phagocytosis (39) and can readily translocate between membrane leaflets depending on pH and charge neutralization of the phosphate head group (33). Whether it accumulates on the surface of sea urchin coelomocytes in association with natSpTrf proteins is not known. PA binding by rSpTrf-E1 may represent the ability of this protein to bind lipids, proteins, and PAMPs with the common attribute of exposed phosphates. However, this does not rule out the possibility of receptors for natSpTrf proteins on small phagocytes and vesicle membranes. If exposed phosphates are a common binding target for a subset of natSpTrf proteins and are present on foreign target cells including LPS and PA, it may be possible for some natSpTrf proteins to bind both bacteria and coelomocytes, which may link bacteria with immune cells through natSpTrf multimerization, thus promoting phagocytosis. Furthermore, if PA clustering is induced by natSpTrf protein multimerization on the coelomocyte surface, this may also aid in driving membrane curvature and endocytosis or phagocytosis.

The extraordinary protein diversity of the natSpTrf proteins that has been reported for sea urchins (14–16) suggests that subsets of these proteins may engage in different levels of phospholipid (or exposed phosphate) binding based on their amino acid sequence compositions. Depending on the element patterns of the mature proteins and putative editing of the mRNAs [reviewed in Ref. (6, 8)], the number of positively charged amino acids varies greatly among these proteins. Consequently, some natSpTrf proteins may not bind to free phosphates on lipids or other molecules, others may bind to different categories of lipids perhaps including the series of phosphatidyl inositols that are phosphorylated at all combinations of sites on the inositol ring. The results presented here infer more complex biological processes for this immune response protein family in sea urchins than previously considered, particularly if each natSpTrf protein variant has multiple and overlapping binding targets that includes not only a range of PAMPs but also a subset of macromolecules with free phosphates including membrane lipids.

## Author Contributions

CML was involved in all aspects of the research. AB generated the FRET data. RS and SG generated liposomes, were involved with the liposome experiments, and provided confocal microscopy imaging and image processing. LCS supervised and directed the research. CML, RS, SG, and LCS wrote, edited, and revised the manuscript. All authors approved the submitted manuscript.

## Disclaimer

This work was prepared while SG was employed at George Washington University. The opinions expressed in this article are the author’s own and do not reflect the view of the National Institutes of Health, the Department of Health and Human Services, or the United States government.

## Conflict of Interest Statement

The authors declare that the research was conducted in the absence of any commercial or financial relationships that could be construed as a potential conflict of interest.

## Footnotes

^1^http://imagej.nih.gov/ij/.

^2^http://gerald-boehm.de/download/cdnn.

^3^http://dichroweb.cryst.bbk.ac.uk/html/home.shtml.

## Supplementary Material

Movie S1 **Giant unilamellar vesicles (GUVs) show fusion, budding, and invagination in the presence of recombinant SpTransformer protein, rSpTransformer-E1 (rSpTrf-E1)**. The movie is composed of a series of confocal microscopy images captured every 15 s starting at ~12 min after the addition of rSpTrf-E1 to the upper right edge of the well that likely diffuses across the well from upper right to lower left. The GUVs show budding (first white arrow that appears), fusion, and invagination (subsequent white arrows) in the presence of rSpTrf-E1.

## Abbreviations

*SpTrf*, transformer genes from the sea urchin *Strongylocentrotus purpuratus*; SpTrf, transformer proteins from the sea urchin *Strongylocentrotus purpuratus*; HeTrf, Trf proteins from the sea urchin species *Heliocidaris erythrogramma*; natSpTrf, native SpTrf proteins; Ni-natSpTrf, nickel-isolated natSpTrf proteins; rSpTrf-E1, recombinant transformer protein with an E1 element pattern; rSpTrf-E1-FITC, biotinylated rSpTrf-E1 labeled with NeutrAvidin fluorescein; rSpTrf-E1-2PA, rSpTrf-E1 bound to two PA lipids; Gly-rich region, the glycine-rich region that is located at the N-terminus of the mature natSpTrf proteins; rGly-rich fragment, the recombinant glycine-rich fragment of rSpTrf-E1; His-rich region, the histidine-rich region that is located near the C-terminus of the mature natSpTrf proteins; rHis-rich fragment, the recombinant Histidine-rich fragment of rSpTrf-E1; rC-Gly, the recombinant fragment that is located at the C-terminal end of the glycine-rich region of rSpTrf-E1; BSA-FITC, biotinylated bovine serum albumin labeled with NeutrAvidin fluorescein; SUV, small unilamellar vesicle; GUV, giant unilamellar vesicle; LUV, large unilamellar vesicle; dextran-488, dextran labeled with Alexa Fluor^®^ 488; PA, phosphatidic acid; NBD-PA, 1-oleoyl-2-(6-[(7-nitro-2-1,3-benzoxadiazol-4-yl)amino]hexanoyl)-sn-glycero-3-phosphate; PC, phosphatidylcholine; TFE, trifluoroethanol; IDP, intrinsically disordered protein; IDR, intrinsically disordered region.
