# Supplementary material for: The Recombinant Sea Urchin Immune Effector Protein, rSpTransformer-E1, Binds to Phosphatidic Acid and Deforms Membranes
Source: Front Immunol. 2017 May 12;8:481. doi: 10.3389/fimmu.2017.00481 (PMC5427130; doi:10.3389/fimmu.2017.00481)
Supplement: Supplementary file 2 [file Image_1.PDF]

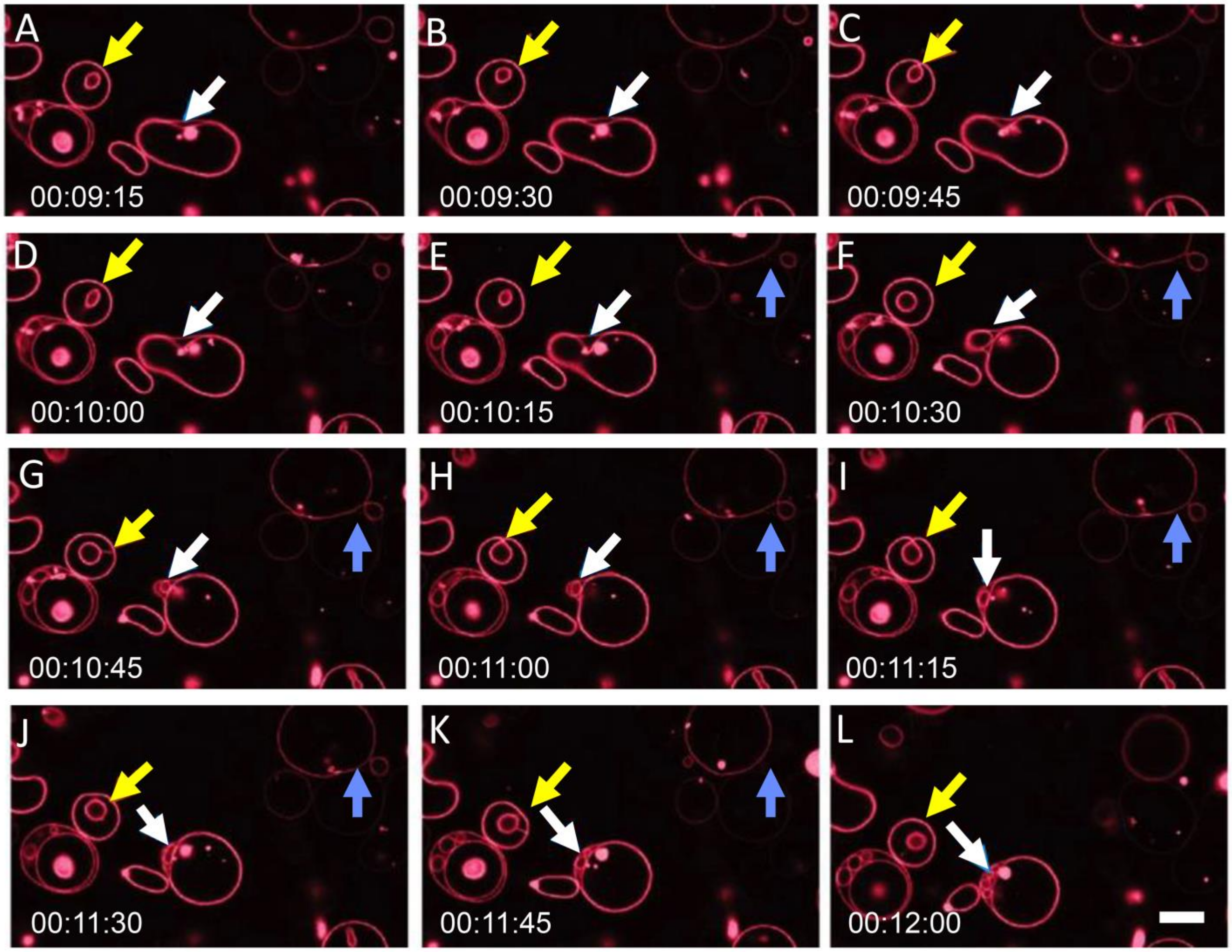

Figure S1. GUVs fuse and invaginate when incubated with rSpTrf-E1-FITC. GUVs composed of 10%PA:PC and labeled with DiD (red) settled to the bottom of 96 well plate for at least 30 minutes before the addition of rSpTrf-E1. Images of GUVs were captured by confocal microscopy every 15 seconds (time is indicated at the bottom left of each panel). GUVs show membrane deformation events such as invagination (white and yellow arrows) and fusion (blue arrows). An elongated GUV (**A**; white arrow) transforms into a kidney bean shape (**C-E**) and eventually invaginates (**E, F**). This newly invaginated GUV eventually invaginates again forming smaller vesicles associated with the membrane (**I-L**; white arrows). Two GUVs of different sizes and in close proximity with each other show fusion (**E**; blue arrows). These two GUVs interact (**F-J**) and the smaller GUV disappears (**K**) suggesting fusion with the larger GUV. In addition to fusion, a multi-laminar GUV (**A-L**; yellow arrows) shows interactions between the inner and outer GUVs through the formatinon of a lipid tube between them (**G, K**). The scale bar corresponds to 20 microns.
