# Supplementary material for: The Recombinant Sea Urchin Immune Effector Protein, rSpTransformer-E1, Binds to Phosphatidic Acid and Deforms Membranes
Source: Front Immunol. 2017 May 12;8:481. doi: 10.3389/fimmu.2017.00481 (PMC5427130; doi:10.3389/fimmu.2017.00481)
Supplement: Supplementary file 3 [file Image_2.PDF]

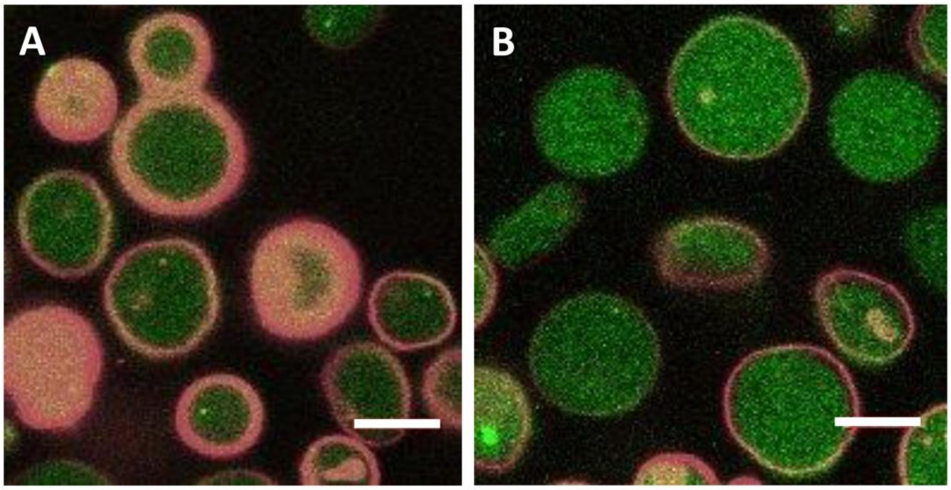


**Figure S2. GUVs do not show changes in morphology in the absence of rSpTrf-E1.** (**A**, **B**) Two independent analyses show that GUVs loaded with dextran-488 (green) plus DiD (red) in the lipid bilayer remain spherical without observable changes in liposome morphology after 30 minutes. These images complement those shown in Figure 3 in the main manuscript. Scale bars indicate 10 microns.
