# Supplementary material for: The Recombinant Sea Urchin Immune Effector Protein, rSpTransformer-E1, Binds to Phosphatidic Acid and Deforms Membranes
Source: Front Immunol. 2017 May 12;8:481. doi: 10.3389/fimmu.2017.00481 (PMC5427130; doi:10.3389/fimmu.2017.00481)
Supplement: Supplementary file 4 [file Image_3.PDF]

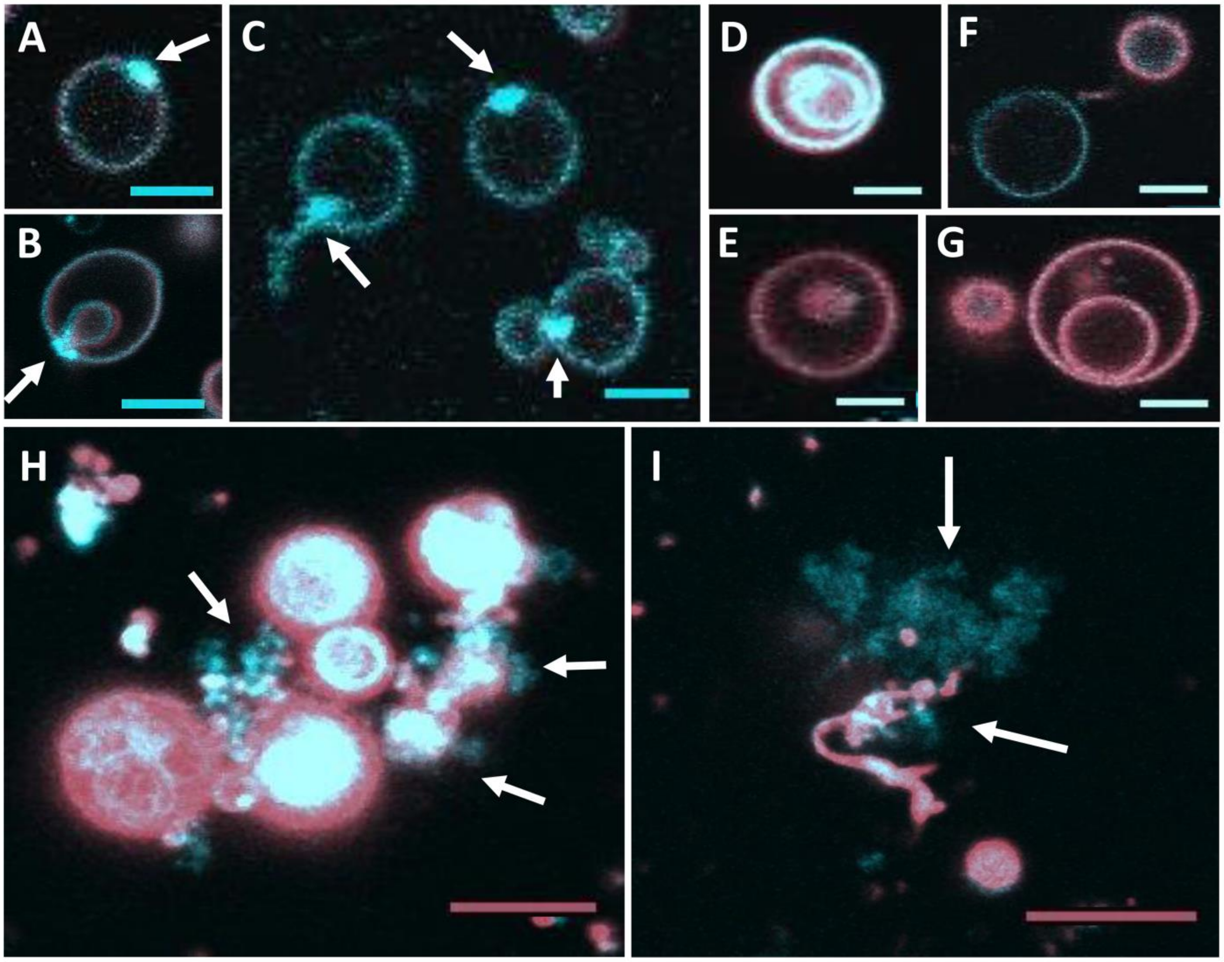

Figure S3. NBD-PA is clustered and separated from GUVs after incubation with rSpTrf-E1. (**A**-**C**) GUVs show clusters of NBD-PA (blue) in the lipid bilayers 20 minutes after the addition of 10 µM rSpTrf-E1 (arrows). (**D**-**G**) GUVs in the absence of rSpTrf-E1 show an even distribution of NBD-PA in the lipid bilayer after 20 minutes. (**H**-**I**) GUVs incubated with 10 µM rSpTrf-E1 for 2 hours show disordered clusters of NBD-PA (arrows) that are separated from the liposomes. Scale bars indicate 10 microns.
